# Cornea verticillata caused by Fabry diseaseCórnea verticilata por doença de Fabry

**DOI:** 10.1590/S1679-45082014AI2901

**Published:** 2014

**Authors:** Ana Luiza Fontes de Azevedo Costa, Victor Roisman, Thiago Gonçalves dos Santos Martins

**Affiliations:** 1 Hospital Federal dos Servidores do Estado, Rio de Janeiro, RJ, Brazil.; 2 Universidade de São Paulo, São Paulo, SP, Brazil.; 3 Universidade Federal de São Paulo, São Paulo, SP, Brazil.

**Figure 1:**
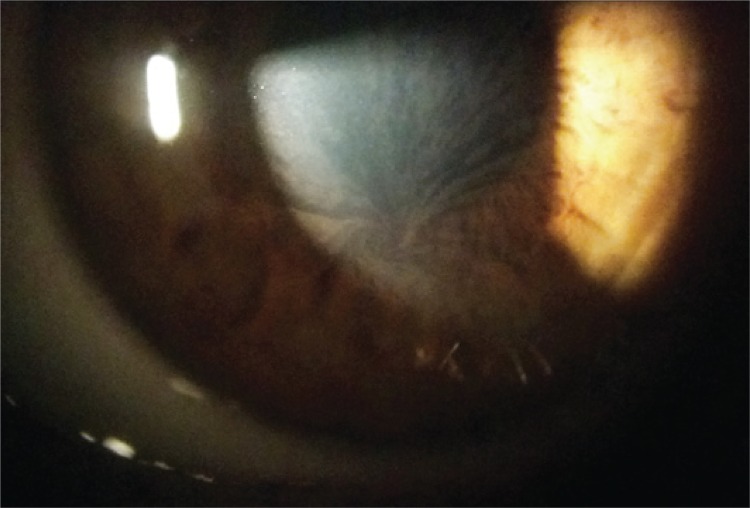
Biomicroscopy showing the corneal verticillata

A 33-year-old woman who works as a yoga teacher and lived in the city of Rio de Janeiro (RJ) - Brazil, sought ophthalmologic care at a routine examination. She did not report comorbidities and previous or regular use of medicines of any type. At examination the patient had good corrected visual acuity of 20/20 in both eyes. Biomicroscopy showed bilateral corneal verticillata, and in the rest of examination no other abnormalities were seen.

Fabry disease is hereditary and X-chromosome linked. This disease is caused by deficiency of the enzyme α-galactosidase A that leads to progressive accumulation of lipid substances in the interior of the endothelium and smooth muscles of blood vessels so that affecting several organs.^(1) ^Men are more affected by Fabry disease because they had the X chromosome to codify the enzyme α-galactosidase. Heterozygote women are commonly the carriers of the Fabry gene (GLA gene, the only gene identified so far), which present a random activity of the enzyme α-galactosidase A that turns its dosage less reliable for the diagnosis. The genetic molecular test is the most reliable diagnostic method for Fabry in female carrier.^(1)^

Angiokeratoma, acroparesthesia, hypertrophic cardiomyopathy, anhidrosis and corneal verticillata are common features of Fabry disease. The disease’s clinical course is heterogeneous and variable especially in women.^(2,3)^

Corneal verticillata was the most found ophthalmology change in men an women with Fabry disease. It presents an incidence of 76.9% in men and 73.1% in women and was describe almost as pathognomonic.^(2,3)^ Initial lesion observed in cornea is diffuse subepithelial layer opacity that progressively adopt a verticillata appearance.^(1) ^This appearance could be an isolated finding without other ocular abnormalities.^(2) ^Overall, there is no impair in vision associated with this corneal alteration.^(3)^

The Fabry disease along with amiodarone therapy is the most common cause of this form of corneal opacity, and the simple history assessment of medication used by the patient helps to clarify the cause.^(4)^

The patient blood sample was sent to a Germany laboratory for molecular genetic test to confirm the diagnosis of Fabry disease. Our patient was probably heterozygote for Fabry disease because, up to the time of examination, she did not have any symptom of the disease expected the corneal alteration. However, in this case, the ophthalmologic investigation was essential to establish the diagnosis and give the patient the opportunity to understand risks and care needed to all sufferers of this rare condition.
