# Supplementary material for: Cornea verticillata caused by Fabry diseaseCórnea verticilata por doença de Fabry
Source: Einstein (Sao Paulo). 2014 Oct-Dec;12(4):527–8. doi: 10.1590/S1679-45082014AI2901 (PMC4879926; doi:10.1590/S1679-45082014AI2901)
Supplement: Translation

# Translation: Córnea verticilata por doença de Fabry

**Authors:** Ana Luiza Fontes de Azevedo Costa, Victor Roisman, Thiago Gonçalves dos Santos Martins

**Affiliations:** 1 Hospital Federal dos Servidores do Estado, Rio de Janeiro, RJ, Brasil.; 2 Universidade de São Paulo, São Paulo, SP, Brasil.; 3 Universidade Federal de São Paulo, São Paulo, SP, Brasil.

**Figura 1:** Córnea verticilata observada à biomicroscopia

Paciente do gênero feminino, 33 anos, professora de ioga, natural e residente da cidade do Rio de Janeiro (RJ), procurou atendimento oftalmológico para exame de rotina. Negava comorbidades bem como uso regular prévio ou atual de qualquer medicação. Apresentava melhor acuidade visual corrigida de 20/20 em ambos os olhos. À biomicroscopia, observava-se córnea verticilata bilateral, sem outras alterações no restante do exame.

A doença de Fabry é hereditária e está ligada ao cromossomo X. Consiste na deficiência da enzima alfa-galactosidase A, que leva ao acúmulo progressivo de substâncias lipídicas no interior do endotélio e músculos lisos de vasos sanguíneos comprometendo, assim, vários órgãos.^(1)^ Acomete mais comumente homens, que só possuem um cromossomo X para codificar a enzima alfa-galactosidase. Mulheres heterozigotas são normalmente carreadoras do gene da doença de Fabry (GLA, até então o único gene identificado), possuindo atividade variada da enzima alfa-galactosidase A, o que torna sua dosagem pouco confiável para o diagnóstico nesses casos. O teste molecular genético é o mais confiável para o diagnóstico de Fabry em mulheres carreadoras.^(1)^

Angioceratoma, acroparestesias, cardiomiopatia hipertrófica, anidrose e córnea verticilata são as típicas manifestações da doença de Fabry. Seu curso clínico é heterogêneo e variado, especialmente em mulheres.^(2,3)^

A córnea verticilata foi a alteração oftalmológica mais encontrada em homens e mulheres com Fabry, com incidência de 76,9% nas mulheres e de 73,1% nos homens, sendo descrita como quase patognomônica.^(2,3)^ A lesão inicial observada na córnea é uma opacidade difusa na camada supepitelial que, progressivamente, adota o aspecto de verticilata.^(1)^ Pode ser um achado isolado sem outras anormalidades oculares.^(2)^ Normalmente, não há baixa visual associada a essa alteração.^(3)^

Juntamente do tratamento com amiodarona, a doença de Fabry é a causa mais comum dessa forma de opacidade corneana, e a simples avaliação do histórico de uso de medicações ajuda a esclarecer a causa.^(4)^

Diante da suspeita, a amostra sanguínea da paciente foi enviada a um laboratório alemão, para teste molecular genético, para a confirmação do diagnóstico. Podemos inferir que a paciente provavelmente é heterozigota para a doença de Fabry, já que, até então, não apresentou sintoma algum da doença, sendo apenas observada a alteração corneana. Apesar disso, a investigação oftalmológica, neste caso, foi essencial para que o diagnóstico fosse estabelecido e que a paciente pudesse estar ciente dos riscos e cuidados necessários a todos os portadores dessa condição rara.
